# Phospholipase A2 is involved in galactosylsphingosine-induced astrocyte toxicity, neuronal damage and demyelination

**DOI:** 10.1371/journal.pone.0187217

**Published:** 2017-11-02

**Authors:** Cedric Misslin, Maria Velasco-Estevez, Marie Albert, Sinead A. O’Sullivan, Kumlesh K. Dev

**Affiliations:** Drug Development, School of Medicine, Trinity College Dublin, Dublin, Ireland; Rutgers University, UNITED STATES

## Abstract

Krabbe disease is a fatal rare inherited lipid storage disorder affecting 1:100,000 births. This illness is caused by mutations in the *galc* gene encoding for the enzyme galactosylceramidase (GALC). Dysfunction of GALC has been linked to the toxic build-up of the galactolipid, galactosylsphingosine (psychosine), which induces cell death of oligodendrocytes. Previous studies show that phospholipase A2 (PLA2) may play a role in psychosine induce cell death. Here, we demonstrate that non-selective inhibition of cPLA2/sPLA2 and selective inhibition of cPLA2, but not sPLA2, also attenuates psychosine-induced cell death of human astrocytes. This study shows that extracellular calcium is required for psychosine induced cell death, but intracellular calcium release, reactive oxygen species or release of soluble factors are not involved. These findings suggest a cell autonomous effect, at least in human astrocytes. Supporting a role for PLA2 in psychosine-induced cell death of oligodendrocytes and astrocytes, the results show inhibition of PLA2 attenuates psychosine-induced decrease in the expression of astrocyte marker vimentin as well as myelin basic protein (MBP), myelin oligodendrocyte glycoprotein (MOG) and the neuronal marker SMI-32 in organotypic slice cultures. These findings provide further mechanistic details of psychosine-induced death of glia and suggest a role for PLA2 in the process. This work also supports the proposal that novel drugs for Krabbe disease may require testing on astrocytes as well as oligodendrocytes for more holistic prediction of pre-clinical and clinical efficacy.

## Introduction

Krabbe disease is a rare inherited lipid storage disorder resulting in oligodendrocyte cell death and subsequent loss of myelin. Krabbe disease is associated with mutations in the *galc* gene encoding for the enzyme galactosylceramidase (GALC) [1] resulting in the build-up of one particular galactolipid, called psychosine, which is suspected to be responsible for the pathology of this illness [2]. Most studies on Krabbe disease have focused on the toxicity of psychosine on oligodendrocytes. Several pathways have been associated with psychosine-induced toxicity including TNFα and IL6 [3] inducible nitric oxide synthase [4, 5] protein kinase C (PKC) [6], NFkB [7], cytochrome c and caspases 3 and 9 [7, 8]

The toxic effects of psychosine have also been attributed to the generation of lysophosphatidylcholine through phospholipase A2 (PLA2) activation [9]. The secretory (sPLA2) and cytosolic (cPLA2) isoforms, are both part of the PLA2 family. The sPLA2 isoform is characterized by a low molecular weight (13–15 kDa) and needs high mM levels of Ca2+ for both binding and catalysis of its substrates [10], compared to the cPLA2 isoform which requires lower concentrations of Ca2+ (μM range) [11]. cPLA2 might be regulated by many cellular processes such as, IL-1 and TNF-α [12, 13], phosphorylation cascades [14, 15] or intracellular Ca2+ elevations [16].

Recently, we have shown in astrocyte cultures and organotypic slice cultures, that psychosine causes astrocyte toxicity and demyelination and that these toxic effects can be attenuated by the sphingosine 1-phosphate receptor (S1PR) agonists pFTY720 and BAF312 [17, 18]. Additionally, psychosine has been shown to induce inflammation in astrocytes via down-regulating the AMP-activated protein kinase (AMPK) activity [19]. Astrocytes are critical in the homeostasis of the CNS, by producing extracellular matrix proteins [4, 20], maintenance of the blood-brain barrier (BBB), transport of metabolites [21–23] and can produce cytokines and chemokines such as IL1, IL6 and IL10, IFNα, IFNβ, CX3CL1, CCL5 and CXCL8 [24]. It has been demonstrated that astrocytes are involved in many neurodegenerative diseases such as multiple sclerosis (MS), Alzheimer’s disease (AD) and Parkinson’s disease (PD) [25, 26]. As such astrocytes play a complex role in the CNS, in both physiological and pathological states.

Having shown previously that psychosine causes astrocyte toxicity that can be attenuated by S1PR drugs [17, 18], here we aimed to examine further the pathways involved in psychosine-induced astrocyte cell death and in this study focussed on the role of phospholipase A2 (PLA2) in this process.

## Materials and methods

### Ethics statement

Astrocytes and organotypic slice cultures from laboratory mice were prepared in strict accordance with the ethical guidelines approved by the institutional care and use committee (IACUC), namely, Trinity College Dublin Ireland, animal research ethics committee (AREC). Mouse euthanasia by decapitation was performed in a manner to minimize suffering of laboratory animals. Human astrocytes from fetal brains were purchased from ScienCell Research Laboratory, USA (1800, Lot Nos. 9063 and 11065), where human tissues have been obtained in compliance with local, state, and federal laws and regulations, as per the ethics outlined by the supplier. Consent to participate is not applicable.

### Cell culture

Human and mouse astrocytes as well as organotypic slice cultures were prepared in accordance with ethical guidelines approved by Trinity College Dublin, Ireland and where relevant as outlined by the supplier ScienCell Research Laboratory, USA. Human and mouse astrocytes, as well as, cerebellar slice cultures were prepared as we have described previously [17, 18, 27–32] Mouse astrocyte purity was >98% also as previously described [27]. Human and mouse astrocytes were serum starved for three hours before treatments. Slices were starved in serum free media for four hours prior to all treatments. Treatments were performed using the following compounds: psychosine (Santa Cruz Biotech, SC-202781), DEDA (Sigma, D8008), Varespladib (Sigma, SML1100), BDDPAA (Calbiochem 525143), Dandrolene (Tocris Bioscience, 0507), which were prepared as 10mM stock solutions dissolved in 100% dimethyl sulfoxide (DMSO, Sigma, D8418); and EDTA (Sigma E5132) which was prepared as 100mM stock solution in sterile water. Concentrations of these drugs were used as suggested by pervious literature [9, 33, 34].

### Immunocytochemistry

Immunocytochemistry of human astrocytes and cerebellar slice cultures, as well as image analysis was performed as described previously [17, 18, 27–32]. The following antibodies were used in this study: Primary antibodies used were: anti-MBP (1:1000; Abcam: ab40390), anti-MOG (1:1000; Millipore: MAB5680), anti-NFH (1:1000; Millipore: MAB5539), anti-Vimentin (Santa Cruz Biotech, sc-373717), anti-Iba1 (1:1000; Wako: 019–1974) and anti-SMI32 (1:1000; Calbiochem NE1023). Secondary antibodies used were: anti-chicken 633 (1:1000; Invitrogen Alexa: A21103), anti-rabbit 488 (1:2000; Invitrogen Alexa: A27034) and anti-mouse Dylight 549 (1:2000; Jackson ImmunoResearch: 715-505-020). The nuclear stain used was Hoechst 34580 (Ivitrogen, H21486). Fluorescence intensity of the confocal images was analysed using ImageJ software (https://imagej.nih.gov/ij/). SMI-32 expression, in the major white matter tracts, was analysed using Imaris® software by calculating the area of expression.

### MTT assay

Astrocytes were plated in 96-well plates and cultured for 24 hours until 70% confluent. The cells were starved with serum free media (DMEM/F12) for three hours, treated with or without compounds, after which the media was removed and replaced by 100μL of fresh, pre-warmed media supplemented with 10μL of 12mM MTT (Invitrogen, M6494). The cells were incubated with MTT for 4 hours at 37°C. After 4 hours of incubation, 75μL of media was removed and 50μL of DMSO per well was added. The cells were incubated for 10 min at 37°C. The plate was mixed and the absorbance read at 540nm.

### Experimental design and statistical analysis

Psychosine was first tested on human astrocytes culture in order to obtain time and concentration curves. For all experiments using astrocytes culture, cells have been starved using serum free media for 3 hours, pre-treated with the specific inhibitor for 1 hour and then treated with psychosine. We also tested our inhibitors in a more complex model using organotypic cerebellar slices culture. Slices were used after 12 days of culture, then treated with psychosine and the inhibitor for 18 hours. The media was changed but treatment with the inhibitor was performed for another 30 hours. Slices were then fixed, stained and visualized using a confocal microscope.

All statistical analysis was performed using GraphPad Prism 5 Software package (GraphPAD Software for Science, San Diego, CA, U.S.A). Unpaired Student’s t-test was used to compare two sets of data from two independent samples, whereas one-way ANOVA was used to compare three or more sets of data from unrelated samples. Newman-Keuls multiple comparison post-hoc test was used after an analysis of variance (ANOVA) for the comparisons of different pairs of mean. Keuls multiple comparison post-hoc, is based on the studentized range distribution, which test all differences among pairs of means. Means were ranked from smallest to largest, and then, the smallest mean was compared to the largest mean. If the test was not significant, then no pairwise tests were significant and no further testing was performed. The significance levels (or alpha levels) were set at p<0.05, p<0.01 and p<0.001.

## Results

### The PLA2 inhibitor DEDA reduces psychosine-induced toxicity in human astrocytes

**Fig 1:**
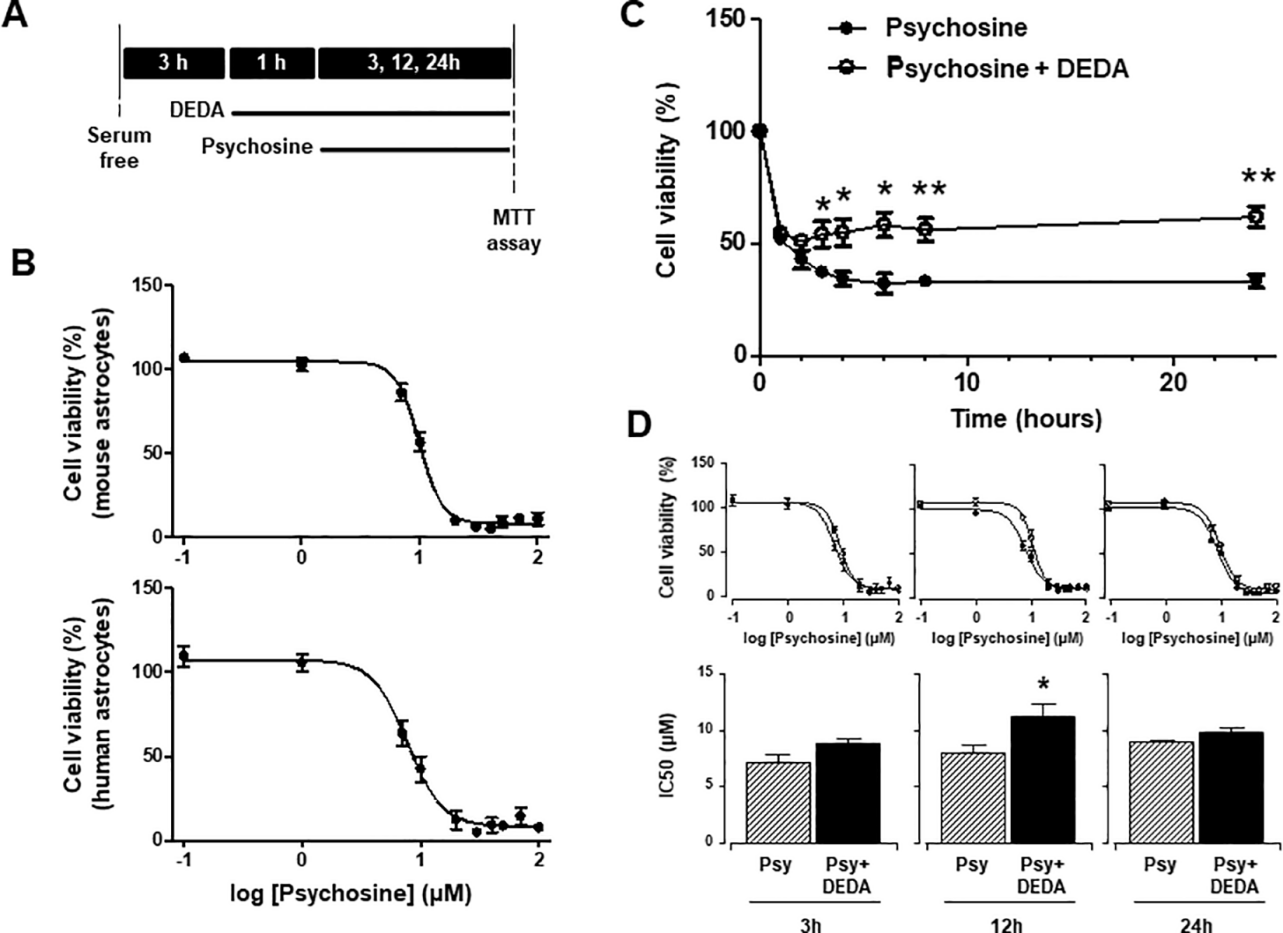
PLA2 inhibitor DEDA protects against psychosine-induced toxicity in human astrocytes. **(A)** Experimental timeline depicting astrocytes were incubated in serum free media for 3h and treated with or without DEDA for 1h before addition of psychosine. Psychosine was used at concentrations indicated for 24h or with 10μM psychosine at incubation times indicated. **(B)** Psychosine induced a decrease in both mouse (*upper graph*) and human (*lower graph*) astrocytes cell viability in a concentration dependent manner (n = 5). **(C)** Human astrocytes were pre-treated with or without the PLA2 inhibitor DEDA (5μM for 1h) before treatment with 10μM psychosine at different incubation times (n = 4). **(D)** Human astrocytes were pre-treated with or without the PLA2 inhibitor DEDA (5μM for 1h) before treatment with different concentrations of psychosine for 3h (n = 3), 12h (n = 4) and 24h (n = 4). Cell viability was quantified by measuring NADP(H) activity using an MTT assay (Vybrant® MTT Cell Proliferation Assay Kit, Life technologies). Graphical data are presented as mean ± s.e.m. Unpaired Student’s t-test *p<0.05, **p<0.001 comparing psychosine ± DEDA.

We have previously described the effects of psychosine on astrocyte cell cultures as well as demyelination in organotypic slice cultures [17, 18]. Here we confirm these findings by showing psychosine decreases cell viability of human (7.7 ± 1.1μM) and mouse astrocytes (9.9 ± 1.0μM) with similar IC50 values (Fig 1B). We can observe an acute drop in cell viability between 1–4 h, with a stable surviving population between 4–24 h (1h: 53.0 ± 2.2%; 2h, 42.9 ± 4.1%; 24h, 33.3 ±3.0%; mean±s.e.m) (Fig 1C). Given that PLA2 plays a role in psychosine toxicity of human oligodendrocytes cell lines [9], we examined next if the same held true for astrocytes. The pre-treatment of human astrocytes with the sPLA2/cPLA2 inhibitor DEDA (5μM) for 1 hour before psychosine (10μM) reduced the decrease in human astrocyte cell viability in a time-dependent manner (2h: 42.9 ± 4.1% vs 51.0 ± 1.3%; 6h: 32.2 ± 4.5% vs 58.4 ± 5.6%; 24h: 33.3 ± 3.0% vs 61.9 ± 4.6%) (Fig 1C). Notably, these effects of DEDA increased the IC50 of psychosine-induced astrocyte cell death at 12 hours (IC50, 7.9 ± 1.1μM vs 10.8 ± 1.1 μM), although not at 3 hours or 24 hours (Fig 1D). These results corroborate our findings that psychosine induces astrocyte cell death and previous findings that PLA2 plays a role in psychosine induced cell death.

### Psychosine-induced changes in human astrocyte morphology and is partially reversed by DEDA

**Fig 2:**
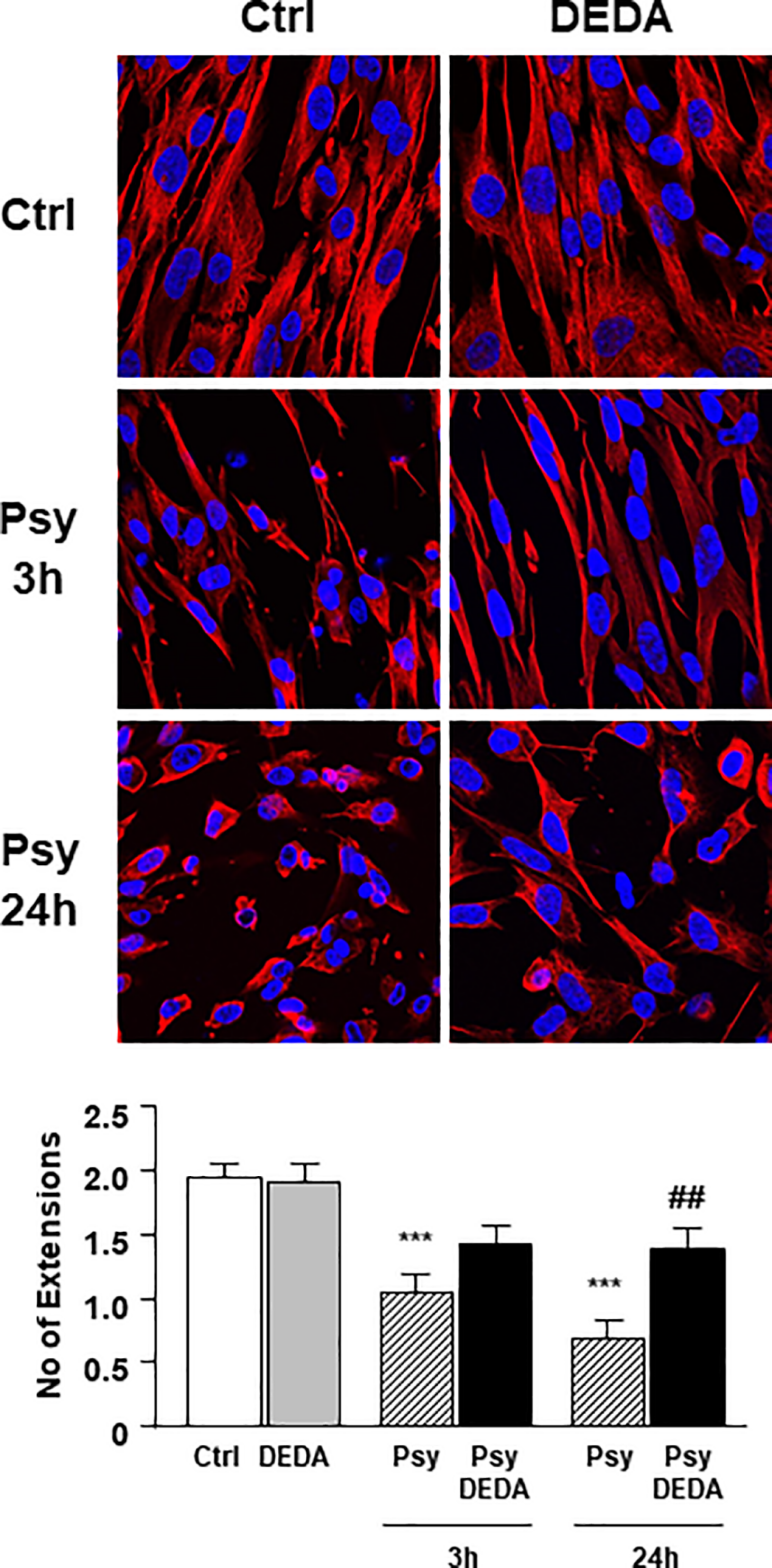
Psychosine-induced changes vimentin in human astrocytes and is partially reversed by DEDA. Human astrocytes were pre-treated with the PLA2 inhibitor DEDA (5μM for 1h) before treatment with psychosine (10μM for 3h or 24h). Representative confocal images displaying Hoesth (blue) and vimentin (red) staining under treatment conditions indicated. A 50μm concentric circle from the nucleus was drawn and the number of cellular extension beyond this were counted. A total of 20–30 cells were counted for each condition. Graphical data are presented as mean ± s.e.m. One-way ANOVA and Newman-Keuls Multiple Comparison post-test, compared to control (*p<0.05, **p<0.01, ***p<0.005) and compared to psychosine treatment (#p<0.05, ##p<0.01, ###p<0.005).

To confirm the protective effect of DEDA on psychosine-induced toxicity we examined the effect of psychosine on the type III intermediate filament astrocyte marker vimentin, which is involved in cytoskeleton formation in astrocytes. Treatment of human astrocytes with psychosine (10μM) for 3 hours or 24 hours induced a reduction of vimentin expression in astrocytes, particularly in the cell processes, suggesting a deregulation of cellular cytoskeleton (Ctrl: 1.03 ± 0.14; 24h: 0.67 ± 0.15) (Fig 2). This change likely reflected the rounding of astrocytes before detachment induced by psychosine. Importantly, these changes in vimentin localization induced by psychosine were reduced by 1 hour pre-treatment with DEDA (5μM) (3h: 1,40 ± 0,15 vs 1.03 ± 0,14; 24h: 1,40 ± 0,14 vs 0.67 ± 0.15) (Fig 2). Overall, these results support the idea that altered cytoskeleton structure may precede psychosine induced cell death and that DEDA attenuates these effects, suggesting the involvement of PLA2.

### Inhibition of cPLA2, but not sPLA2, partially rescues human astrocytes from psychosine-induced toxicity

**Fig 3:**
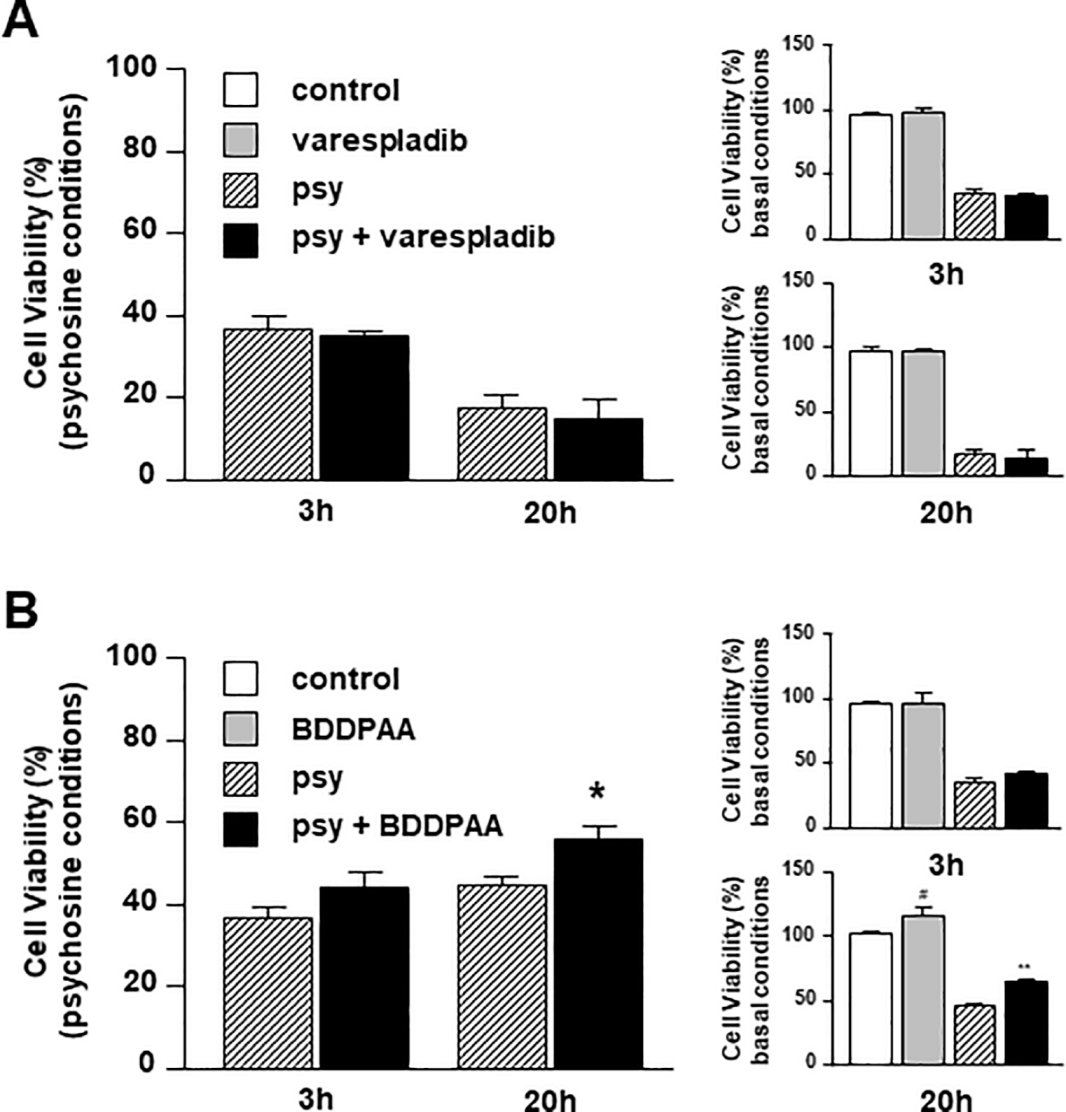
cPLA2 inhibitor BDDPAA, but not sPLA2 inhibitor Varespladib, attenuates psychosine-induced toxicity in human astrocytes. Human astrocytes were pre-treated with **(A)** the specific sPLA2 inhibitor Varespladib (10μM for 1h) (n = 4) or **(B)** the specific cPLA2 inhibitor BDDPAA (1μM for 1h) (n = 4–5) before treatment with psychosine (10μM for 3h or 20h). Cell viability was quantified by an MTT assay. Data is presented as mean + s.e.m. The main graph shows the ratio between control vs psychosine and drug vs drug+psychosine and smaller graphs show comparisons to vehicle control and psychosine treatment.

Given that DEDA is a non-selective sPLA2/cPLA2 inhibitor, we examined the effects of Varespladib (sPLA2 inhibitor) and BDDPAA (cPLA2 inhibitor) on psychosine-induced toxicity in human astrocytes. Notably Varespladib has reached clinical trials for acute coronary syndrome, although not proven to be efficacious [35]. Human astrocytes were pre-treated with Varespladib (10μM) or BDDPAA (1μM) for 1 hour before psychosine (10μM). Varespladib did not significantly alter the number of astrocytes under basal conditions (3h: 96.1 ± 1.7% vs 97.0 ± 5.0%; 20h: 97.4 ± 2.0% vs 97.0 ± 1.6%) nor showed protective effects on psychosine-induced astrocyte cell death (3h: 36.3 ± 3.3% vs 34.7 ± 1.2%; 20h: 16.9 ± 3.5% vs 14.2 ± 5.2%) (Fig 3A). In contrast, BDDPAA increased modestly the number of human astrocytes compared to control in basal conditions after 20 hours treatment (3h: 96.1 ± 1.7% vs 96.8 ± 7.0%; 20h: 101.2 ± 2.8% vs 116.0 ± 5.8%) and showed a further significant protection against psychosine (beyond its basal effect) at that time-point (3h: 36.3 ± 3.3% vs 43.6 ± 4.1%; 20h: 44.4 ± 2.4% vs 55.5 ±3.3%) (Fig 3B). These results, demonstrate that the cPLA2, but not sPLA2, may play a role in psychosine-induced toxicity in human astrocytes.

### Extracellular calcium is required for psychosine-induced human astrocyte cell death

**Fig 4:**
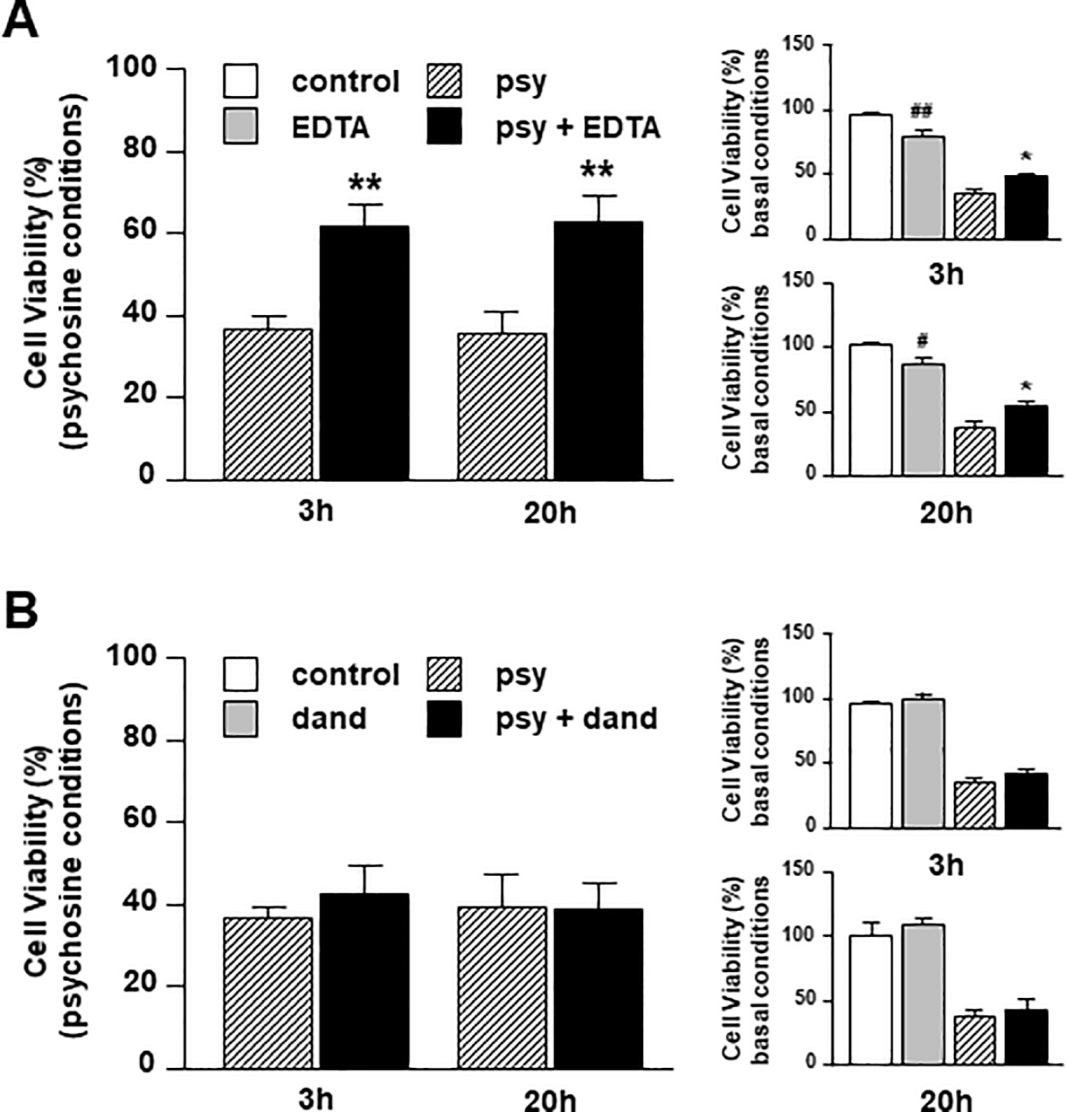
Extracellular, but not intracellular, calcium is required for psychosine-induced human astrocyte cell death. Human astrocytes were pre-treated with **(A)** the calcium chelator EDTA (1mM for 1h) (n = 4–6) or **(B)** the ryanodine receptor antagonist Dandrolene (10μM for 1h) (n = 4) before treatment with psychosine (10μM for 3h or 20h). Cell viability was quantified by an MTT assay. Data is presented as mean + s.e.m. The main graph shows the ratio between control vs psychosine and drug vs drug+psychosine and smaller graphs show comparisons to vehicle control and psychosine treatment.

Previous studies indicate that psychosine causes calcium influx and mitochondrial depolarization, which may precipitate cell death via apoptosis in oligodendrocytes [33]. It is also reasonable to suggest that such calcium influx may also promote PLA2 activation and catalytic activity [36]. To investigate the requirement of calcium in psychosine-induced cell death of astrocytes, we pre-treated human astrocytes with EDTA (1mM) (to chelate extracellular calcium) or Dandrolene (10μM) (a ryanodine receptor antagonist that inhibits the release of calcium from intracellular stores) 1 hour before treatment with psychosine (10μM). EDTA reduced modestly the number of human astrocytes under basal conditions, (3h: 96.1 ± 1.7% vs 80.2 ± 5.7%; 20h, 101.6 ± 1.2% vs 86.3 ± 5.5%) but still protected significantly against psychosine-induced toxicity (3h: 36.3 ± 3.3% vs 61.5 ± 5.9%; 20h: 35.3 ± 5.8% vs 62.8 ± 6.1%) (Fig 4A). In contrast, Dandrolene did not alter the number of human astrocytes under basal conditions (3h: 96.1 ± 1.7% vs 98.7 ± 4.0%; 20h: 100.1 ± 9.9% vs 108.2 ± 3.9%) and had no protective effect against psychosine toxicity (3h: 36.3 ± 3.3% vs 42.5 ± 6.9%; 20h: 38.8 ± 8.7% vs 39.1 ± 6.2%) (Fig 4B). It appears therefore that extracellular calcium influx, but not intracellular calcium release plays an important role in psychosine induced astrocyte cell death.

### Psychosine induced astrocyte cell death does not involve production of ROS or the release of a toxic soluble factors

**Fig 5:**
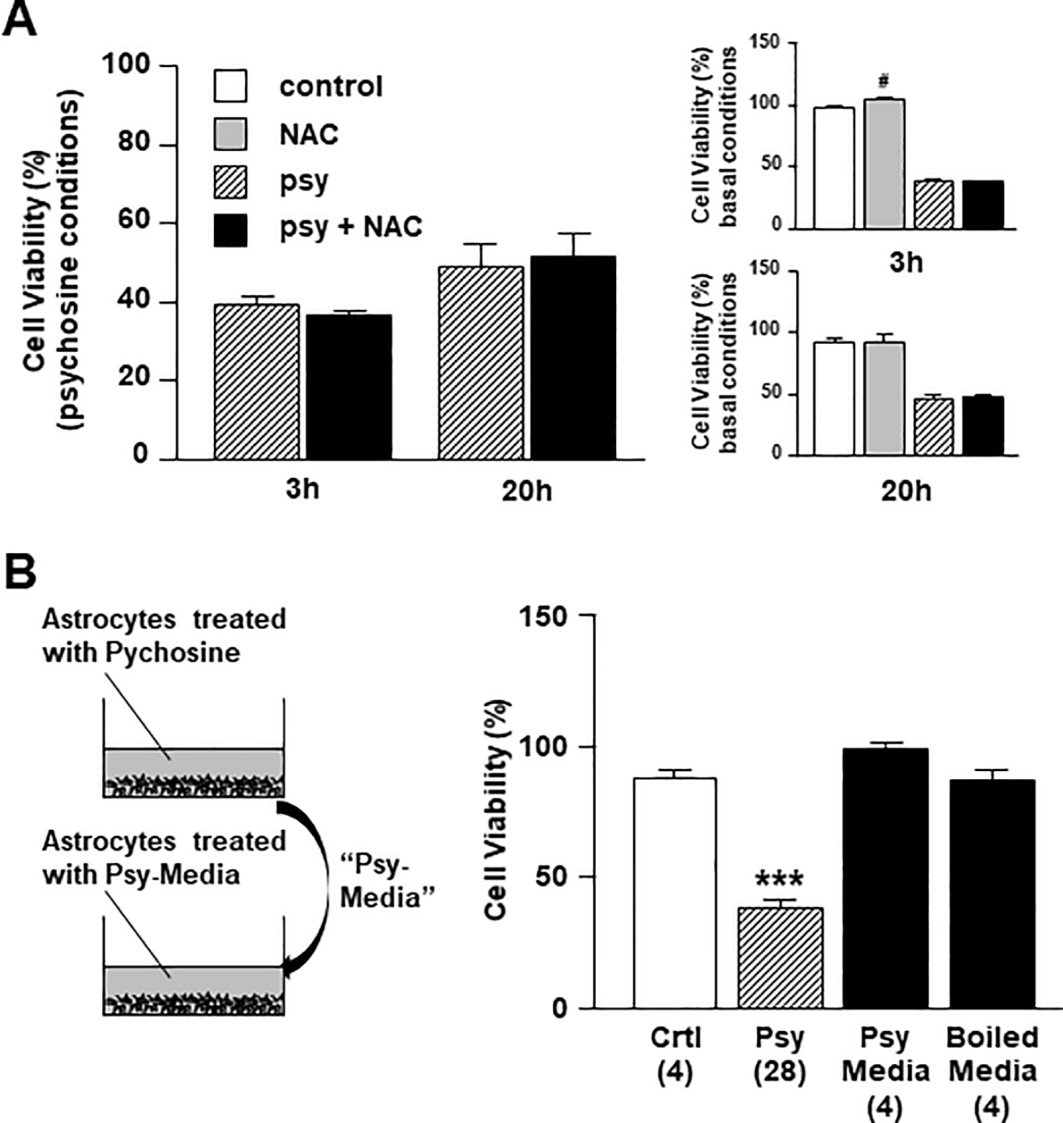
ROS production or extracellular mediators are not involved in psychosine-induced toxicity in human astrocytes. (A) Human astrocytes were pre-treated with a specific ROS inhibitor N-acetylcysteine (NAC) (1mM for 1h) (n = 3–4) before treatment with psychosine (10μM for 3h or 20h). The main graph shows the ratio between control vs psychosine and drug vs drug+psychosine and smaller graphs show comparisons to vehicle control and psychosine treatment. (B) Human astrocytes were serum starved for 3h before treated with psychosine (10μM for 1h). After washing three times with serum free media to wash out the added psychosine, fresh complete media was incubated for 5h with the treated astrocytes and this conditioned media (“psy-media”) was then added to naive human astrocytes and incubated for 20h. One-way ANOVA and Newman-Keuls Multiple Comparison post-test, compared to control (*p<0.05, **p<0.01, ***p<0.005). The number of experiments is indicated in the parenthesis (n = 4–28). Cell viability was quantified by an MTT assay. Data is presented as mean + s.e.m.

Psychosine induces reactive oxygen species (ROS) in the oligodendrocyte MO3.13 cell line [33]. We thus investigated if pre-treatment with a specific ROS inhibitor (N-acetylcysteine, NAC) (1mM) 1 hour before psychosine (10μM) had protective effects on astrocyte viability, but found no obvious rescue (3h: 39.3 ± 1.8% vs 36.1 ± 1.2%; 20h: 48.9 ± 5.9% vs 51.6 ± 5.7%) (Fig 5A). To further examine the mechanism by which psychosine induces astrocyte cell death, we investigated if human astrocytes treated with psychosine may release toxic mediators into the media that can induce cell death in a cell non-autonomous manner. Human astrocytes treated with psychosine (10μM) for one hour were washed free from psychosine and fresh media was added. After five-hour incubation, the conditioned media (“psy-media”) was collected, boiled at 90°C for 5 min, and/or directly incubated with naive human astrocytes for 20 hours. While psychosine induced significant human astrocyte cell death compared to control (37.8 ± 2.8% vs 87.3 ± 3.0%), no cell death was induced by the psy-media (98.7 ± 1.9 vs 87.3 ± 3.0%) or by the boiled psy-media (Fig 5B). These results suggest that psychosine induced astrocyte cell death does not involve production of ROS or the release of a toxic soluble factors, and that psychosine toxicity occurs in a cell autonomous manner, at least in isolated astrocyte cell cultures.

### Inhibition on PLA2 attenuates psychosine-induced decrease in the astrocyte marker vimentin

**Fig 6:**
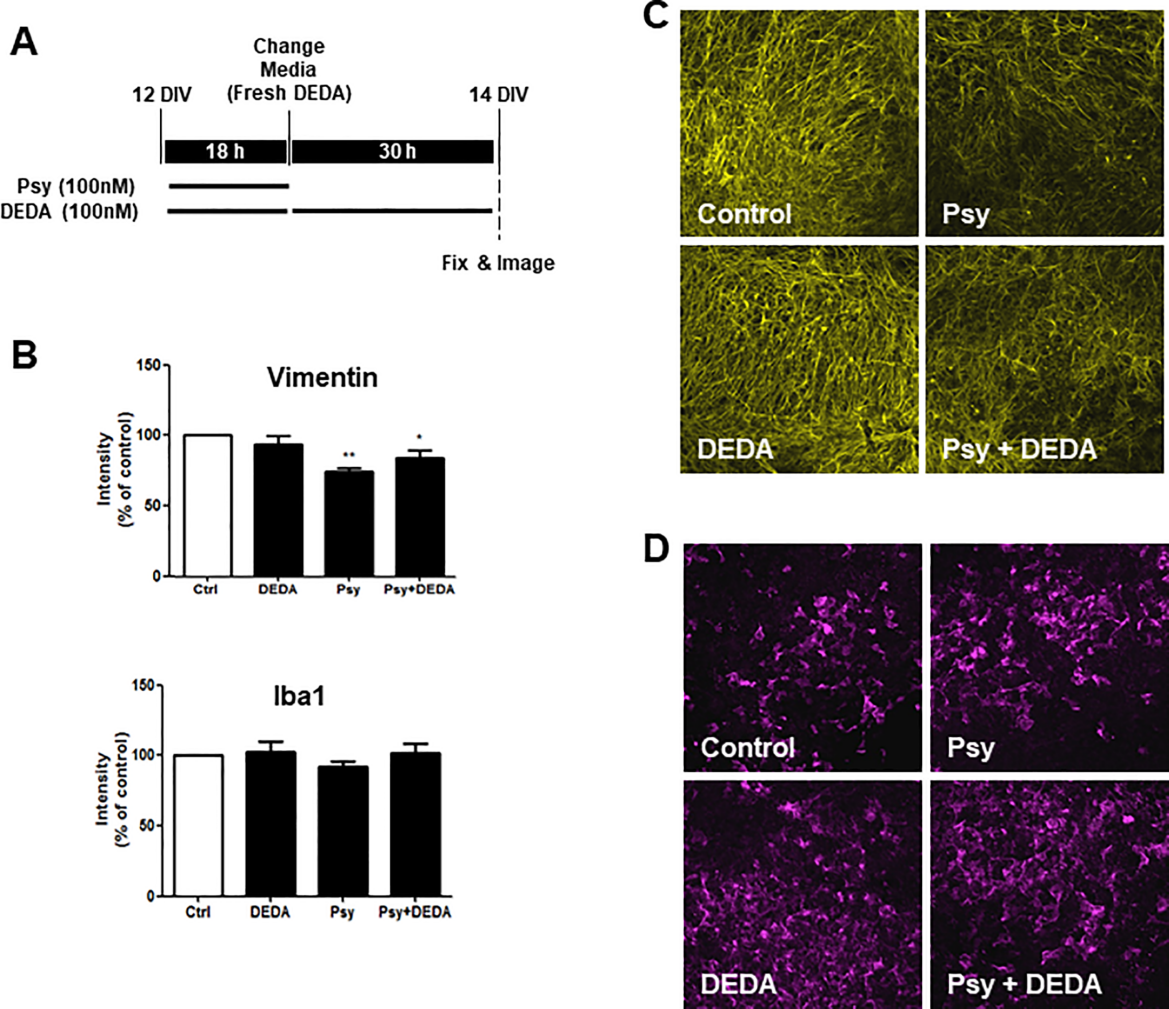
DEDA inhibits psychosine-induced loss of vimentin expression in cerebellar slices. **(A)** Organotypic slice cultures were prepared from the cerebellum of P10 mice and grown in culture for 12 days. Slices were treated with psychosine (100nM) and/or DEDA (100nM) for 18 h. The medium was then changed and DEDA treatment continued for a further 30 h, or control medium was added for this time. Cerebellar cultures were then processed for immunocytochemistry. **(B)** Bar graphs illustrate changes in vimentin and Iba1 staining after psychosine (100 nM) ± DEDA (100 nM) treatments. **(C)** Representative confocal images displaying the astrocyte marker vimentin (yellow) and **(D)** the microglia marker Iba1 (purple) immunostaining under treatment conditions indicated. Confocal images captured at ×10 magnification. Confocal images were taken at 10x magnification; scale bar 200μm. Mean fluorescence was calculated using a total of 25–36 independent ROI observations in each experiment. Data are presented as mean ± SEM. (n = 8), one-way ANOVA and Newman–Keuls multiple comparison post-test *p<0.05; #p<0.05 comparing control ± psychosine.

In our previous studies we have shown that psychosine induces demyelination in organotypic slice cultures [17, 18]. Thus, to further these previous studies and examine whether our isolated astrocyte culture data held true in a more complex cellular system, we next examined the effects of psychosine on expression of the astrocyte marker vimentin, using these brain slice cultures. For the first time, we show that psychosine reduces the expression of vimentin in cerebellar slices (74.38 ± 7.13% vs 100%) (Fig 6B and 6C). This data is in agreement with our current astrocyte culture studies data and furthers our previous findings, suggesting psychosine reduces cell viability of astrocytes. Importantly, in these experiments, DEDA attenuated the decrease in expression of vimentin caused by psychosine (83.50 ± 16.45% vs 74.38 ± 7.13%) (Fig 6B and 6C). We have also reported psychosine does not regulate the expression levels of the microglia marker Iba1 (ionized calcium binding adapter molecule 1), although it should be noted Iba1 is not a specific marker of altered microglia reactivity. Here we confirmed that psychosine does not alter the expression of Iba1 (92.13 ± 10.43% vs 100%) and that DEDA had no effects on the expression of this marker either (102.1 ± 23.27% vs 100%) (Fig 6B and 6C).

### Psychosine-induced demyelination is reversed by the inhibition of PLA2

**Fig 7:**
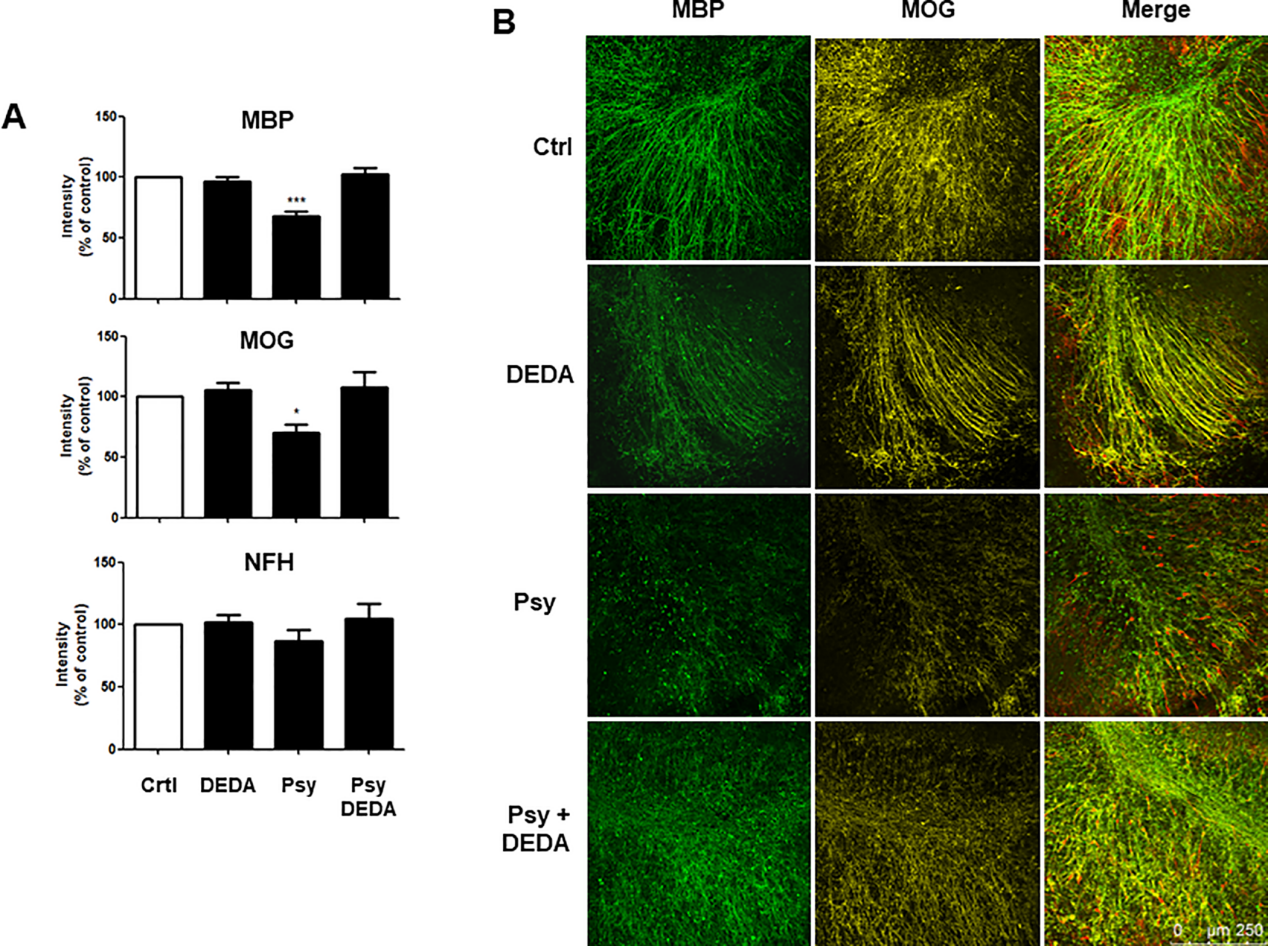
DEDA treatment inhibits psychosine-induced demyelination of cerebellar slices. **(A)** Bar graph illustrating changes in MBP, MOG and NFH staining after psychosine (100 nM) ± DEDA (100 nM) treatments. **(B)** Representative confocal images displaying MBP (MBP, green), MOG (MOG, yellow) and neurofilament (NFH, red) immunostaining under treatment conditions indicated. Confocal images captured at ×10 magnification; scale bar 200μm. Mean fluorescence was calculated using a total of 25–36 independent ROI observations in each experiment. Data are presented as mean±s.e.m. (n = 7), one-way ANOVA and Newman–Keuls multiple comparison post-test *p<0.05; #p<0.05 comparing control ± psychosine.

A recent study has identified a pathogenic phenotype of reactive astrocytes found in a number of neurodegenerative and neuroinflammatory disorders. These neurotoxic cells can directly induce neuronal and oligodendroglial death in addition to loss of their homeostatic function [37]. We have also shown previously that psychosine induced astrocyte cell death in dissociated cell cultures and demyelination in organotypic slice cultures can be rescued by the S1PR drugs pFTY720 and BAF312, both of which are efficacious in multiple sclerosis [17, 18]. Here we aimed to confirm psychosine-induced demyelination in organotypic cerebellar slices and to examine the role of PLA2 in this complex brain slice model system. Organotypic cerebellar slices were exposed to psychosine (100 nM) in the presence or absence of DEDA (100 nM) for 18 h and treated for a further 30 h with DEDA (100 nM) or control medium (Fig 7A). In agreement with our previous studies [17, 18], psychosine induced a modest decrease in the levels of neurofilament H (NFH) (86.38 ± 25.82% vs 100%), with significant demyelination compared with control, as observed by expression of myelin basic protein (MBP) (67.88 ± 10.82% vs 100%) and myelin oligodendrocyte glycoprotein (MOG) (70.17 ± 17.19% vs 100%) (Fig 7B). Importantly, this psychosine-induced demyelination was significantly attenuated by DEDA (100 nM) (MBP: 102.3 ± 15.44% vs 67.88 ± 10.82%; MOG: 108.0 ± 30.52% vs 70.17 ± 17.19%) (Fig 7B). These results further support the idea that PLA2 plays a role in psychosine-induced cell toxicity and may have a multimodal effect in both astrocytes and oligodendrocytes.

### Axonal damage induced by psychosine is reversed by the inhibition of PLA2

**Fig 8:**
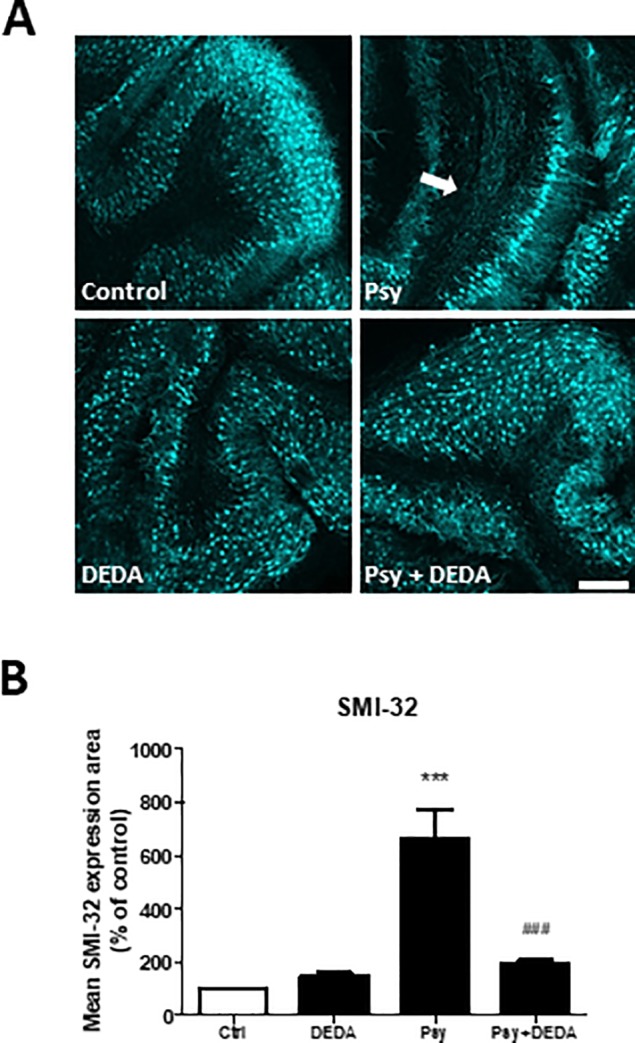
DEDA treatment attenuated the psychosine-induced axonal damage. Relative surface area of SMI-32 axonal expression **(B)** shows a significant increase in the white matter tracts induced by psychosine treatment, and attenuated by DEDA co-treatment. **(A)** Representative images of SMI-32 expression in cerebellar slice culture. Confocal images taken at 10x magnification; scale bar 200μm. Graphs expressed as mean±s.e.m. (n = 6), repeated measures one-way ANOVA, Newman-Keuls post-hoc test performed. ***p<0.0001 compared to control, ^###^p< 0.0001 compared to psychosine treatment.

Neurofilament H is a structural cytoskeletal protein specific to neurons [38] comprising phosphorylated and non-phosphorylated epitopes of NFH [39]. It has been previously shown that non-phosphorylated NFH epitopes, such as SMI-32, are mainly expressed by purkinje and neuronal cell bodies under physiological conditions, with minimal expression in the axonal tracts [40, 41]. More interestingly, an increase in the expression of SMI-32 in the axonal tracts has been associated with disturbances in axonal transport, demyelination and neuronal damage in multiple sclerosis [42, 43]. In our previous studies we have utilised SMI-32 as a marker for neuronal damage and have shown an increase in the expression of SMI-32 in the white matter tracts under demyelinating GOX-CAT treatment in mouse cerebellar slice culture [30]. In agreement with that, and to further our previous findings [17][18], here we report an increase of SMI-32 in the white matter tracts under psychosine treatment (Fig 8A). Importantly, we find that inhibition of sPLA2 by DEDA attenuated this increment in SMI-32 (193% ± 8.81% vs Psy: 593.3% ± 81.6%, ^###^p<0.0001). Notably, DEDA did not induce the expression of SMI-32 in the axonal tracts by itself compared to control (136.2% ± 11.47% vs 100%) (Fig 8B).

## Discussion

### Summary of findings

Recent studies in our laboratory have demonstrated that psychosine induces cell toxicity in astrocytes [17, 18]. In the current study, we investigated whether PLA2, calcium and ROS pathways are involved in this process. Results demonstrate that PLA2 inhibitors rescue the decrease of human astrocyte cell viability induced by psychosine treatment. We show that the cytosolic isoform of PLA2 (cPLA2), rather than the secretory form (sPLA2), is involved in psychosine-induced toxicity in astrocytes. We also find that extracellular calcium is required for psychosine-induced toxicity, whereas limiting intracellular calcium has little effect. Our experiments show that ROS generation does not play a significant role in psychosine-mediated astrocyte cell death. In addition, the data show that media obtained from astrocytes treated with psychosine does not cause further astrocyte cell death, suggesting that psychosine induces toxicity in a cell-autonomous manner. Using a more complex cellular system, we find that psychosine inhibits expression of vimentin in organotypic slice cultures and these effects are attenuated by the PLA2 inhibitor DEDA, corroborating our dissociated astrocyte cell culture findings. We find also that PLA2 attenuates psychosine-induced decrease in the expression of myelin basic protein (MOG) and myelin oligodendrocyte glycoprotein (MOG) in organotypic slice cultures. Lastly, we demonstrate that axonal damage, as measured by the expression of SMI-32 in the axonal tracts, induced by psychosine was attenuated under inhibition of PLA2. Taken together, our results suggest that psychosine modifies astrocyte as well as oligodendrocyte function, likely leading to subsequent axonal damage and that the PLA2 signalling pathway plays a role in this process.

### The cPLA2 isoform plays a role in psychosine-induced toxicity in astrocytes

The PLA2 inhibitor DEDA has a protective effect on psychosine-induced cell death in oligodendrocytes [9]. Activation of PLA2 leads to the generation of arachidonic acid and metabolites, which are involved in processes such as inflammation, apoptosis, cell growth and differentiation [44, 45]. Arachidonic acid leads to the activation of p38 MAPK and transcription factors such as AP1 and NFκB [46]. The metabolism of arachidonic acid also gives rise to ROS, which may induce oxidative stress [47]. The arachidonic acid (and/or lysophosphatidylcholine, LPC) signalling pathway(s) likely result in the loss of oligodendrocytes, where inhibitors of PLA2, such as DEDA, can rescue psychosine-induced cell death [9]. Previous studies have demonstrated that astrocytes release arachidonic acid (with phospholipase A2 involvement) under ROS conditions [48, 49]. We note however, no previous research, to our knowledge, has investigated psychosine-induced levels of arachidonic acid or LPC in astrocytes. A caveat in our current study is we also did not investigate if psychosine changes levels of LPC and/or AA. Here, our data suggested that DEDA itself promoted moderately the number of astrocytes and more importantly protected these cells from psychosine toxicity. Given that DEDA may inhibit both sPLA2 and cPLA2, and also 5-lipoxygenase and formation of leukotrienes, we also used specific PLA2 inhibitors. The low molecular weight (13–15 kDa) sPLA2 requires high concentrations of Ca2+ (mM range) for activity [10, 11], whereas cPLA2 is larger (85 kDa) and requires a lower concentration of Ca2+ (μM range) [50]. Here, we used Varespladib, a sPLA2 inhibitor with an IC50 of 9.6nM [35] and BDDPAA, a cPLA2 inhibitor, with an IC50 of 1.8nM [51]. Our results showed that the cPLA2 inhibitor BDDPAA, but not the sPLA2 specific inhibitor Varespladib, attenuated psychosine-induced toxicity, at least in astrocytes. These results are in contrast to previous studies showing that sPLA2 plays a role in psychosine-induced toxicity in oligodendrocytes [9] and highlights possible mechanistic differences between cell types. It should be noted that we also observed that BDDPAA treatment by itself modestly increased the number of human astrocytes. Arachidonic acid has been shown to act as a second messenger and regulate apoptosis [44, 52]. Inhibiting cPLA2 activity may lead to a decrease in arachidonic acid and a loss of cell ability to produce lipid mediators [53, 54]. This hypothesis could explain why inhibiting cPLA2 using BDDPAA has a modest increase in cell number.

### Extracellular calcium signalling is required for psychosine-induced toxicity in human astrocytes

Previous studies in oligodendrocytes have shown that psychosine leads to a rapid calcium peak after 5–8 minutes treatment and followed by a slower second peak that precedes cell death [33]. It has been demonstrated that the chelation of extracellular calcium extends oligodendrocyte cell survival and reduces mitochondrial ROS production [33]. Here, we used EDTA to chelate extracellular calcium and the ryanodine antagonist Dandrolene to inhibit release of calcium from intracellular stores. The data showed that EDTA, but not Dandrolene, significantly rescued psychosine- induced cell death of human astrocytes. This protective effect was observed to be more pronounced than the PLA2 inhibitors and lasted both after short and long periods of psychosine treatment. Our data suggest that, similar to oligodendrocytes, psychosine might cause a calcium deregulation, which also plays a role in astrocyte cell death.

### ROS is not thought to be involved in psychosine-induced toxicity in astrocytes

In addition to deregulating PLA2 and/or calcium signalling, psychosine might induce the production of intra-mitochondrial ROS production [33]. This ROS production might be associated with changes in calcium signalling, in particular, intra-mitochondrial calcium signalling [55]. With regard to PLA2, the over-production of arachidonic acid from PLA2 enzymatic activity might also lead to ROS production [56]. We used the ROS inhibitor N-acetylcysteine (NAC), which limits ROS generation via inhibiting c-Jun N-terminal kinase, p38 MAP kinase, redox-sensitive activating protein-1 and NFκB [57, 58]. In contrast with oligodendrocytes, our data show that inhibiting ROS production did not affect human astrocyte cell survival when treated with psychosine. Oligodendrocytes are particularly sensitive to oxidative stress [59], where the production of ROS is damaging more so to oligodendrocytes, compared to astrocytes or microglia [60]. Therefore, in agreement with our findings, ROS inhibitors may have protective effects on psychosine-mediated oligodendrocyte cell death, while not so in astrocytes.

### Psychosine mediates astrocyte cell death in a cell-autonomous manner

Others and we, have shown that psychosine does not alter the levels of cytokines in astrocytes [5, 17, 18]. However, psychosine can enhance the production of cytokines such as TNFα, IL8 or MCP1 in addition to LPS in peripheral blood mononuclear cell (PBMC) form patients [3], and stimulates the cytokine-mediated production of nitric oxide (NO) in C6 glial cell line [5]. Based on these findings, we investigated whether psychosine-induced toxicity in astrocytes is associated with the release of toxic mediators into the media and/or if psychosine-induced toxicity occurs in a cell-autonomous manner. To investigate this hypothesis, healthy human astrocytes were incubated with media from astrocytes that had been pre-treated with psychosine. Our data showed that this conditioned media did not cause cell toxicity when exposed to healthy human astrocytes. This data suggests that psychosine-mediated astrocytes cell death does not involve the release of mediators into the extracellular space and therefore occurs in a cell-autonomous manner.

### Psychosine deregulates astrocytes and oligodendrocytes in concert

Many studies have shown that psychosine causes demyelination and that the dysfunction of oligodendrocytes is associated with Krabbe disease. To support the idea that psychosine also deregulates astrocytes we examined the effect of this toxin on markers of all three glia cell types in a more intact cell culture model using organotypic slice cultures. In agreement with our previous studies [17, 18], psychosine reduced the expression of MBP and MOG suggesting demyelination, while showing no effect on the microglia marker Iba1. For the first time, here we show that psychosine reduced expression of the astrocyte marker vimentin. Importantly, DEDA attenuated the psychosine-induced decrease in expression of MBP and MOG as well as vimentin supporting the idea that astrocyte function as well as oligodendrocyte myelination state is altered in Krabbe disease. Furthermore, we note that the increased expression of SMI-32 in the axonal tracts–a marker of axonal damage and demyelination- induced by psychosine is attenuated by DEDA treatment.

## Conclusion

This study was initially designed to investigate whether the signalling pathways involved in psychosine-mediated cell death of oligodendrocytes are shared in astrocytes, with a focus toward the role of PLA2. For the first time, we show here that PLA2 and calcium signalling is involved in psychosine-induced toxicity in astrocytes, similar to oligodendrocytes. We confirm that astrocytes are responsive to psychosine treatment in a cell autonomous manner and during psychosine-induced demyelination. These findings provide new mechanistic details psychosine-mediated cell death of glial cells and suggest that targeting both oligodendrocytes and astrocytes for therapeutic intervention may be preferential. We conclude that testing PLA2 inhibitors in the twitcher mouse model may be highly desirable and positive outcomes may pave a way toward a clinical trial for such drugs in Krabbe disease.
